# Study of intestinal parasitic infections associated with HIV infection in Douala, Cameroon

**DOI:** 10.1186/1742-4690-9-S1-P48

**Published:** 2012-05-25

**Authors:** Leopold G Lehman, Lafortune Kangam, Eveline Nguepi, Marthe-Lilianne Mbenoun, Charles F Bilong Bilong

**Affiliations:** 1 University of Yaoundé I, Douala, Cameroon

## Aim

Gastrointestinal infections are common in people living with HIV. Diarrhea occurred in 30 to 90% of them. However reports on the prevalence of gastrointestinal parasites (GIP) and HIV infection are very few in Cameroon. The purpose of this study was to assess the GIP prevalence in the context of increasing availability of highly active antiretroviral therapy (HAART).

## Materials and methods

A prospective study was conducted from January to December 2011 wherein stool and blood samples were collected from 201 patients in 3 hospitals of Douala. The stool samples were stained with Kinyoun and Safranin to identify coccidian oocysts. Saline preparations were used to identify ova, cysts and larva. Preparations were observed under UV light microscope CyScope® (Partec Görlitz, Germany). CD4+ T lymphocytes were counted with a flow cytometer CyFlow® (Partec Görlitz, Germany). Chi-square test was used for statistical analyses and P-value <0.05 was considered significant.

## Results

The global prevalence of intestinal parasites was 27.8%. Seventeen (48.6%) out of 37 patients with diarrhea and 38 (23.2%) out of 164 without diarrhea were parasitized. The most frequent parasites were Candida spp. (13.2%), Cryptosporidium spp. (7.4%) and Entamoeba histolytica/dispar (3%). A significant correlation (p=0.002) was observed between the presence of parasites and diarrhea. The highest parasite counts (p=0.035) and diarrhea (p<0.0001) were found in patients with CD4+ < 200 cells/µl. Srongyloïdes stercoralis, Trichuris trichuira and Isospora belli were only found in diarrheal sample.

## Conclusions

The overall prevalence of GIP is decreasing in Douala, probably due to the growing avaibility of HAART. This study highlights the importance of looking for intestinal parasite in HIV patient with low immunity presenting with diarrhea in Douala, which is not the case in our hospitals.

